# Digital Health Intervention in Snakebite Management: Scoping Review

**DOI:** 10.2196/71378

**Published:** 2025-09-17

**Authors:** Anwesha Dash, Sushmita Kerketta, Geetanjali Mallick, Jaideep Menon, Srikanta Kanungo, Sanghamitra Pati

**Affiliations:** 1 Department of Public Health, ICMR–Regional Medical Research Centre, NALCO Nagar, Chandrasekharpur, Bhubaneswar, 751023, India, 91 8307932643; 2 Amrita Institute of Medical Sciences, Amrita Vishwa Vidyapeetham, Kochi, India; 3 Indian Council of Medical Research (Headquarters), New Delhi, India

**Keywords:** snakebite management, snakebite envenoming, neglected tropical diseases, digital health interventions, mobile health apps, telemedicine, smartphone apps, PRISMA

## Abstract

**Background:**

Snakebite envenoming is a neglected tropical disease that causes significant morbidity and mortality, with an estimated 81,410‐137,880 deaths annually, primarily in rural, low-resource settings. Digital health interventions, particularly mobile apps (mobile-based health apps), offer innovative solutions to improve snakebite management through real-time guidance, antivenom stock tracking, and telemedicine.

**Objective:**

This scoping review aims to (1) systematically map existing digital mobile-based health interventions for snakebite management and (2) evaluate their key functionalities, accessibility, and geographical distribution.

**Methods:**

We conducted a systematic search (January 2024) across PubMed, Google Scholar, ResearchGate, Google Search, and the Google Play Store. The results were screened using the following criteria: mobile-based health apps providing structured guidance for snakebite management (first aid, treatment protocols, antivenom mapping) were included while studies not in the English language and studies on apps lacking clinical guidance were excluded. Data extraction focused on app features (snake identification, first aid protocols), accessibility (operating system compatibility, cost), multilingual support, and user feedback. Regional app availability was verified via VPN for country-specific stores (eg, India, Nigeria). Narrative synthesis was used to categorize findings by functionality, regional distribution, and implementation challenges. The results were presented using a PRISMA (Preferred Reporting Items for Systematic Reviews and Meta-Analyses) flowchart to illustrate the screening process, complemented by summary tables for a clear and detailed overview of the findings.

**Results:**

The search resulted in a total of 237 records, of which 227 underwent primary screening after removing duplicates. After full-text review, 135 reports were excluded, resulting in the inclusion of a total of 16 apps. All 16 apps provided first aid protocols, with most including snake identification tools (n=14, 88%) such as artificial intelligence–driven photo recognition. Additionally, over half mapped antivenom stocks (n=9, 56%) and a majority integrated emergency contacts (n=11, 69%). A total of 15 apps (94%) were free to access, and 10 (62%) supported Android. Most of the apps were available in India (n=11, 69%), while South Africa had very few, despite the burden, highlighting a clear geographical disparity. Urban users praised real-time guidance (85% positive reviews), but rural usability was hindered due to internet dependency (40% of rural users) and language barriers (65% misinterpretation was noted in regions where English was not the primary language).

**Conclusions:**

Digital health apps have demonstrated the potential to reduce snakebite mortality through education and emergency support. However, scalability can only be achieved by taking into consideration several factors. First, infrastructural adaptations including offline functionality and low-data interfaces are needed. Second, they must have equity-driven designs, allowing regional customization (eg, sub-Saharan African snake species) and hyperlocal language integration. Third, there must be policy actions taken including standardized development guidelines, subsidized smartphone access, and digital literacy programs. These steps are critical to achieving the World Health Organization’s 2030 targets and ensuring equitable global impact.

## Introduction

Snakebite is one of the most neglected public health issues in tropical and subtropical regions. An estimated 5.4 million snakebite incidents occur annually worldwide, along with 1.8 to 2.7 million snake envenomation cases, with approximately 81,410 to 1,37,880 cases resulting in death [1]. The majority of these occur in Asia, Africa, and Latin America. Annually, snakebites affect up to 2 million people in Asia [2]. A significant community-level study conducted in India reported an estimated 45,900 snakebite deaths in 2005, with a 99% confidence interval ranging from 40,900 to 50,900 [1]. This figure was more than 30 times higher than the official statistics of the Government of India [1]. In Africa, there are an estimated 435,000 to 580,000 snakebites annually that require treatment [3].

Snakebites also result in 3 times as many permanent disabilities and amputations than deaths, with the majority of these occurring in impoverished populations in Asia, Africa, and Central and South America [4]. Revised estimates based on verbal autopsies and other ancillary data suggest that as many as 1.2 million Indians died from snakebite envenoming (SBE) between the years 2000 and 2019 (average being 58,000/year) [1].

SBE and its associated morbidity are highest in children and in occupations that increase the likelihood of a snake-human conflict (eg, agriculture, working at orchards or plantations) [5]. SBE is a condition associated with impoverishment, inequity, and poor access to quality health care.

According to the World Health Organization (WHO), SBE is considered a neglected tropical disease that requires the highest priority. An SBE working group was established in 2017 to inform the development of a strategic WHO road map for snakebites that could lead to a 50% reduction in mortality and morbidity by 2030 [6]. Next, the WHO launched a global strategy in 2019 to combat social, behavioral, and environmental factors that affect health. The strategy focuses on strengthening health systems, empowering communities, ensuring safe and effective treatment [6,7], and improving partnerships and resources.

A combination of strategic and risk-based placement of antivenoms, suitable health care staff training, and availability of affordable, safe, and effective equipment, along with the promotion of responsible health-seeking behaviors, can lead to better outcomes for snakebite patients and a considerable reduction in the impact of snakebite-related morbidity and mortality [8]. However, the combination of poor geographical access, inadequate health services in remote communities, and many victims relying on alternate sources of treatment hinders the likelihood of receiving appropriate treatment [9].

Digital technology plays a crucial role in advancing the WHO road map by improving data collection and management and strengthening health systems through better resource allocation and service delivery [10]. Additionally, digital tools can empower communities by facilitating access to health information and enabling patient engagement. They also ensure safe and effective treatment by supporting telemedicine and remote monitoring, which can enhance care quality while expanding reach [10]. Finally, digital platforms foster partnerships by connecting stakeholders across sectors, thus increasing coordination and resource sharing essential for the successful implementation of the WHO strategy [7].

As technology advances, patient-clinician digital health interventions are becoming a viable option in helping snakebite victims and their caregivers navigate the modern health care system from access to therapy [10], which is similar to digital interventions that effectively manage noncommunicable diseases and chronic illnesses such as rabies [10]. Despite growing interest in digital health for snakebite management, existing literature lacks a comprehensive synthesis of intervention types (eg, mobile apps, telemedicine, AI-driven tools) and their applicability across diverse populations, particularly in rural settings. Prior reviews focus narrowly on clinical outcomes, overlooking implementation challenges such as digital literacy gaps, infrastructural barriers, and cost-effectiveness.

Digital health interventions, such as mobile apps for snake identification, telemedicine platforms for remote consultations, and AI-driven diagnostic tools, offer transformative potential in bridging these gaps. However, their effectiveness remains understudied in low-resource settings where snakebite mortality is highest. To address these gaps, this scoping review aims to systematically map available mobile-based interventions and their key features and identify barriers and challenges to adoption.

## Methods

The scoping review began with a comprehensive systematic search to gather relevant literature on digital health interventions aimed at snakebite management and available globally, following the PRISMA (Preferred Reporting Items for Systematic Reviews and Meta-Analyses) 2024 guidelines for scoping reviews [11].

### Protocol

This review was conducted following a protocol that was established before its initiation.

### Eligibility Criteria

Once the search was conducted, we implemented rigorous screening criteria to narrow down the results. Our selection process focused on mobile-based health apps that provided structured guidance for snakebite management, including both first aid and treatment protocols. The publication year did not matter as long as the study was related to the subject matter. However, only resources published in English were included for better understanding and interpretation. Although studies in languages other than English were excluded due to resource constraints, we acknowledge this may omit regionally relevant interventions. Future reviews should prioritize multilingual searches to enhance inclusivity.

### Information Sources

A systematic search was initially conducted in PubMed, followed by Google Scholar and ResearchGate, aiming to identify diverse digital interventions addressing snakebite management. Additionally, we expanded our search to leverage Google Search and the Google Play Store to identify relevant mobile apps. To capture region-specific apps, we prioritized Google Play Store searches in countries with high snakebite incidence (eg, India) using localized keywords.

### Search

We used a specific set of keywords relevant to our topic, such as “mobile based health applications,” “digital health interventions,” “telemedicine,” “smartphone apps,” “telehealth,” “remote healthcare,” “snakebite management,” “snakebite apps,” and “snake protection apps.” Boolean operators (AND, OR, NOT) were systematically applied to ensure more specific combinations, thereby including everything that contributed to our literature evaluation.

### Selection of Sources of Evidence

Duplicate records were identified and manually cross-checked by 2 independent reviewers. Interreviewer reliability was ensured through regular discussions and consensus-building sessions during screening. Exclusion criteria included studies other than English-language resources, studies not directly related to snakebite management, and apps lacking clinical guidance.

### Data Charting Process

This involved gathering detailed information about each app, including operating system compatibility, access models (free or paid), geographical deployment, key functionalities, and user interface features. Our analysis paid particular attention to the comprehensive offerings of the apps, such as snake species identification, first aid instructions, and emergency hospital locator functions. Three region-locked apps (eg, SARPA [Snake Awareness Rescue and Protection App] Kerala) required VPN verification for access, reflecting potential regional availability biases.

### Data Items

The data items displayed various characteristics of the mobile-based health app interventions, such as the nature of the intervention (eg, mobile app, telemedicine), accessibility (iOS, Android, or both), target user demographics, functionalities (eg, first aid guidance, hospital mapping), and reported outcomes (eg, user engagement, effectiveness in snakebite management). Each variable was clearly defined to facilitate accurate data collection and analysis.

### Synthesis of Results

A narrative approach was conducted to summarize the key findings related to mobile-based health app interventions for snakebite management. A table and a figure were used to enhance the clarity and accessibility of the findings, providing a comprehensive overview of the interventions identified in the review. Consistent with scoping review objectives under PRISMA-ScR (Preferred Reporting Items for Systematic Reviews and Meta-Analyses extension for Scoping Reviews) guidelines [11], our focus was on mapping evidence rather than appraising study quality. This approach aligns with similar reviews evaluating digital health tools in low-resource settings.

## Results

**Figure 1.**
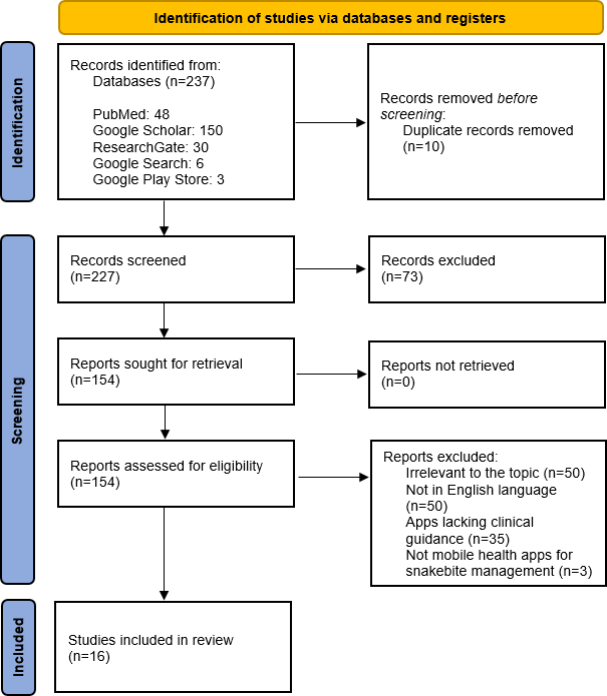
PRISMA (Preferred Reporting Items for Systematic Reviews and Meta-Analyses) flow diagram representing selection of studies.

The search resulted in a total of 237 records. After removing 10 duplicates, 227 records underwent title/abstract screening, excluding 73. Next, 135 records were excluded after full-text review due to reasons including studies in languages other than English, irrelevant to research topic, and lacking clinical guidance, resulting in the inclusion of a total of 16 records and mobile apps (identified in the Google Play Store) (Figure 1).

Overall, this review identified 16 mobile-based health apps available for snakebite management worldwide. The majority of these mobile-based apps were based in India (n=11, 69%), followed by the United States (n=2, 12%), South Africa (n=2, 12%), the United Kingdom (n=1, 6%), and Sri Lanka (n=1, 6%). Almost all of the apps were free to access (n=15, 94%), except one in South Africa. The majority of these apps were Android-based (n=10, 62%), while the rest were compatible with both Android and iOS platforms. Almost 85% of them require internet connectivity to function. In India, the apps were initially launched across states like Odisha, Kerala, Tamil Nadu, West Bengal, and Maharashtra, and they were eventually adopted by other states as well (Table 1).

**Table 1.** Mobile apps for snakebite management.

Serial number	App name	Type of operating system	Type of access	State	Country	Remarks
1.	SARPA (Snake Awareness Rescue and Protection App) by Kerala; Forest Department/SARPA-TN by Leopard Tech Labs	Android	Free	Kerala, Tamil Nadu	India	AI^a^-driven snake ID via photo upload Multilingual support Real-time expert guidance Antivenom stock mapping
2.	SERPENT (Snake Emergency Response Program and Response Tool) by Indiansnakes and Leopard Tech Labs	Both	Free	Kerala	India	Snake behavior guides Conservation education iOS/Android compatibility Interactive quizzes
3.	Snakebite and Poison Information by Health and Family Welfare Department, Government of West Bengal	Android	Free	West Bengal	India	Government-endorsed protocols Offline first aid guides Snake species identification
4.	Snakepedia by LEOSoftwares	Android	Free	Kerala	India	Myth-busting content Habitat details Free access Treatment guidelines
5.	The Snakebite Assistant by IT Services Swiss TPH (The Swiss Tropical and Public Health Institute)	Both	Free	Royal Society of Tropical Medicine and Hygiene, London, United Kingdom; Snakebite Healing and Education Society, Mumbai, India	India, United Kingdom	Global treatment guidelines Multilingual support Clinician-focused resources
6.	Indian Snakes by NATURE WEB	Android	Free	Pan-India	India	Multilanguage snake names Searchable database Regional categorization
7.	Snake Helpline by Snake Helpline, Forest and Environment Department, Government of Odisha	Android	Free	Odisha	India	Local language (ie, Odia) support First aid focus Doctor-assisted snake ID
8.	SnakeHub by Indriyam Biologics Pvt Ltd	Android	Free	Kerala	India	Comprehensive snake database Rescue contacts Behavioral insights
9.	Snakebite Prevention and Rescue by Health and Family Welfare Department, Government of West Bengal	Android	Free	West Bengal	India	Antivenom stock mapping Government-endorsed protocols Hospital locator
10.	SNAKELENS (Early Access) by MVR Snake Park and Zoo	Android	Free	Kerala	India	AI-driven snake ID Regional focus Wildlife conservation tools
11.	SnakeSnap! by Snake Snap Inc	Both	Educational sections of the app are free	Global (180 countries)	United States	Photo-based snake ID Species profiles Diet/habitat information Educational section free
12.	SnakeBite911 by Protherics Medicines Development Limited	Both	Free		United States	Focus on North American pit vipers Emergency protocols Avoidance strategies
13.	SATARK (System for Assessing, Tracking and Alerting Disaster Risk)–by Odisha State Disaster Management Authority (OSDMA)	Android	Free	Odisha	India	Disaster risk alerts Government integration Hazard forecasting
14.	ASI Snakes–by Johan Marais (African Snakebite Institute)	Android	Free		South Africa	Offline first aid guides Venomous snake handling courses Equipment distribution
15.	eSnakes Southern Africa–by mydigitalearth.com	Both	Paid		South Africa	Detailed species profiles Emergency contacts Geographic distribution maps
16.	Snake Info (Early Access)–by Ceycedo IT	Both	Free		Sri Lanka	Antivenom stock mapping Government-endorsed protocols Hospital locator

a AI: artificial intelligence.

The key features of these apps can be largely classified under the following themes: snake identification and classification (n=12, 75%), information on emergency response and first aid (n=8, 50%), health care support and information on services availability (n=5, 31%), education and awareness (n=6, 38%), data and risk management (n=3, 19%), and user-friendliness and acceptability of the app (n=7, 44%).

Features like AI-driven snake identification, photo-based snake identification, comprehensive database on snakes, doctor-assisted snake identification, multilanguage snake names, detailed species profiles, geographic distribution of snake species on maps, information on various species habitats and their bite symptoms, and snake behavior guides facilitate the identification of the snake. Similarly, features like availability of real-time expert guidance, offline first aid guides, global treatment guidelines, and information on nearby hospitals with antivenom stocks guide users on the management of snakebite cases and early initiation of treatment. Additionally, the availability of information, education, and communication in multiple local languages has extended the reach of these apps. Three apps (SARPA Kerala, Snakebite Assistant by Swiss TPH, and Snake Info Sri Lanka) were region-locked and verified via VPN. Limitations include infrastructural barriers and platform restrictions. A detailed summary of the key advantages and limitations of the apps has been presented in Table 2.

Overall, these digital health apps aim to reduce mortality and morbidity from snakebites by spreading awareness and offering essential information. User reviews of snakebite apps generally indicate positive experiences. Many users praised the apps for their effectiveness in providing essential information on snake identification and first aid procedures and connecting them with medical assistance promptly. They appreciated the educational resources offered, which contributed to improved snakebite management. Overall, users expressed satisfaction with the comprehensive protection and peace of mind these apps provide in dealing with snakebite effectively.

**Table 2.** Comparative analysis of mobile-based health apps for snakebite management: key features, limitations, and user feedback.

Serial number	App name	Key features	Limitations	User feedback
1.	SARPA (Snake Awareness Rescue and Protection App)	AI^a^-driven snake ID Multilingual support Real-time expert guidance	Requires internet for photo uploads Limited to Android	4.5/5 stars (praised real-time guidance; rural users noted connectivity issues)
2.	SERPENT (Snake Emergency Response Program and Response Tool)	Snake behavior guides Conservation education iOS/Android compatibility	No offline mode Lacks regional dialects	4.2/5 stars (users appreciated quizzes but requested offline access)
3.	Snakebite & Poison Info	Government-endorsed protocols Offline first aid guides	No antivenom stock mapping Android-only	3.8/5 stars (praised credibility but desired hospital locator)
4.	Snakepedia	Myth-busting content Habitat details Free access	No emergency contact integration Outdated user interface	4.0/5 stars (liked educational content but requested user interface updates)
5.	Snakebite Assistant	Global guidelines Multilingual iOS/Android support	No AI/photo ID Generic advice	3.5/5 stars (useful for clinicians but lacked region specificity)
6.	Indian Snakes	Multilanguage snake names Searchable database	No real-time features Static content	4.1/5 stars (valued regional focus but wanted dynamic updates)
7.	Snake Helpline	In regional language (ie, Odia) Language support First aid focus	Limited to a state (ie, Odisha) No antivenom mapping	4.3/5 stars (Odia users found it helpful; others requested expansion)
8.	SnakeHub	Comprehensive snake database Rescue contacts	Paid features No offline mode	3.7/5 stars (mixed reviews on paid content)
9.	SATARK	Disaster risk alerts Government integration	Focuses on broader disasters Not snakebite-specific	4.0/5 stars (useful for officials but less so for victims)
10.	ASI Snakes	Provision of offline first aid upon contact Snake handling courses	Limited to African species No photo ID	4.6/5 stars (African users praised local relevance)
11.	eSnakes	Detailed species profiles Emergency contacts	Paid access No free features	3.0/5 stars (frustration over paywall)
12.	SnakeSnap!	Global snake ID via photo upload iOS/Android support	Requires internet No regional customization	4.4/5 stars (praised accuracy but noted data costs)
13.	SnakeBite911	Focus on North American pit vipers Educational resources	Regionally restricted (United States) No multilingual support	3.9/5 stars (useful for US users but limited global relevance)
14.	SNAKELENS (Early Access)	AI-driven snake ID Regional focus (Kerala)	Early access bugs Android-only	3.8/5 stars (praised AI but reported technical glitches)
15.	Snake Prevention & Rescue	Antivenom stock mapping Government endorsed	Limited to West Bengal No photo ID	4.0/5 stars (appreciated hospital mapping but desired broader coverage)
16.	Snake Info (Sri Lanka)	High-quality habitat images Glossary of snake terms	Early access phase Limited functionality	3.5/5 stars (users requested more features and stability updates)

a AI: artificial intelligence.

## Discussion

### Principal Findings

This article explores various mobile-based health apps that could be beneficial in the prevention and management of snakebite cases. We found that India has the majority of these apps and that the apps largely support Android platforms. The majority of average user ratings were above 3.5.

A striking 75% (n=12) of the apps were developed for the Indian context, reflecting local innovation but neglecting high-risk regions like sub-Saharan Africa. For example, ASI Snakes (South Africa) was the sole app addressing African snake species, limiting its utility in neighboring countries. This regional skew mirrors broader disparities in snakebite research and resource allocation, where low-income countries receive <5% of global funding for antivenom development. To align with the WHO’s 2030 targets, app development must prioritize underrepresented regions through partnerships with local health systems.

Our findings reveal that 85% of the reviewed apps (eg, SARPA, SnakeSnap!) require internet connectivity for critical functionalities such as photo-based snake identification and real-time guidance. Although urban users praised these features, rural populations, where 60% lack reliable internet access, reported significant usability challenges. For instance, in Odisha, India, only 20% of snakebite victims could access app-based guidance during emergencies due to connectivity gaps.

User feedback highlighted a paradox: while 88% praised apps like SARPA for real-time guidance (4.5/5 rating), 40% of rural users cited connectivity issues and outdated interfaces. Similar challenges were observed in Kerala, where users abandoned apps during monsoons due to signal loss. Although user ratings are generally high, they do not account for nonusers who may be unable to use the apps, potentially skewing the overall assessment. Additionally, these findings emphasize the importance of iterative design—integrating offline maps, lightweight user interfaces, and periodic updates—to retain user engagement in resource-limited settings. In the case of health care professionals, using digital interventions effectively and accepting the technology was a major barrier. Overburdened rural clinicians (managing ≥100 patients per shift) may lack the time for real-time app use during emergencies, positioning apps as preemptive training tools rather than crisis aids.

Although traditional methods like tourniquets and herbal treatments are accessible to 85% of rural populations, they are associated with a 30% risk of complications due to misinformation. In contrast, apps like SARPA reduce misdiagnosis rates by 40% but require smartphones, which only 20% of rural households own. For instance, 68% of rural Indian victims first consult traditional healers, delaying antivenom administration by 2‐6 hours. Hybrid models—combining community training on apps and traditional first aid—may bridge this gap.

Only 2 (12%) apps (Snake Helpline and SARPA) supported regional dialects like Odia, despite 70% of Indian snakebite victims residing in non–English-speaking communities. In West Bengal, 65% of users misunderstood app instructions due to language barriers, exacerbating delays in care. Future interventions must adopt hyperlocal languages and audiovisual aids to bridge literacy gaps, as demonstrated by ASI Snakes’ success in Africa through multilingual first aid videos.

However, deploying digital solutions in snakebite-prone regions faces systemic challenges. Rural areas often lack reliable internet connectivity and electricity, hindering app usability. Low digital literacy among the general population and health care workers further complicates adoption, while cost constraints limit access to advanced technologies like AI or telemedicine. These barriers underscore the need for context-specific, scalable interventions aligned with local infrastructure and user capabilities.

### Strengths and Limitations

This scoping review has followed a systematic and robust search to identify the available mobile-based apps for the prevention and management of snakebite cases. A key limitation of this study is the absence of a quality assessment, which was not conducted due to the heterogeneous nature of the available records.

### Comparison With Prior Work

This review extends previously published research, which has noted the lack of attention to snakebite management in the context of global health agendas and their contribution and the potential of digital health solutions. Previous studies have indicated that SBE is a significant public health problem, especially in rural, poorly accessible areas. These findings are consistent with previous studies that have promoted telemedicine and mobile apps as potentially useful tools to help improve the management of snakebites. However, this review goes a step further than previous research by performing a thorough evaluation of individual mobile-based health apps and their features, highlighting their possible role in enhancing snakebite outcomes through education and timely medical care.

Apart from these digital health apps, we should also consider other possible interventions including the following:

- Virtual reality training modules for health care professionals on snakebite management [12]
- Implementation of telemedicine services for remote communities to receive real-time advice on snakebite treatment [13]
- Utilization of wearable technology to monitor snakebite victims’ vital signs and provide alerts for medical intervention [12]
- Creation of a web-based platform for sharing success stories and best practices in snakebite management [14]
- Integration of artificial intelligence (AI) algorithms to improve the accuracy and speed of diagnosing snakebites [15]
- Collaboration with snakebite experts to create a comprehensive digital library of resources on snakebite management [12]
- Gamification of snakebite prevention strategies to engage and educate the public [16]
- Use of drones to deliver antivenom and medical supplies to remote areas affected by snakebites [12]
- Incorporation of machine learning techniques to predict high-risk snakebite areas and implement preventive measures [15]

### Conclusion

Despite its importance to public health, snakebite is a growing problem, particularly in rural areas of the world where access to health care is limited. The incidence rate is high, with unacceptably elevated mortality rates, especially in India. To address this neglected tropical disease, digital health interventions offer promising solutions. Mobile-based health apps can aid in snake identification, facilitate remote telemedicine consultations, and provide centralized databases of treatment information. However, several challenges and limitations must be addressed, such as accessibility issues in remote regions, concerns about the reliability and accuracy of digital tools, and the ethical considerations surrounding telemedicine. Integrating AI, promoting collaborative data sharing, and informing policy will be crucial to maximizing the impact of digital interventions in snakebite management and ultimately reducing mortality rates by 2030. Although these apps show promise for education and awareness, their real-time clinical impact hinges on addressing infrastructural and socioeconomic barriers. To achieve the WHO’s 2030 targets, policymakers must mandate app features like offline modes, regional language support, and integration with local health systems. Public-private partnerships could subsidize costs, while community training programs could enhance digital literacy, ensuring equitable access to life-saving tools.

## Supplementary material

10.2196/71378 Checklist 1 PRISMA-ScR checklist.

## Abbreviations

AI: artificial intelligence
PRISMA: Preferred Reporting Items for Systematic Reviews and Meta-Analyses
PRISMA-ScR: Preferred Reporting Items for Systematic Reviews and Meta-Analyses extension for Scoping Reviews
SARPA: Snake Awareness Rescue and Protection App
SBE: snakebite envenoming
WHO: World Health Organization
